# Perovskite-based color camera inspired by human visual cells

**DOI:** 10.1038/s41377-023-01072-y

**Published:** 2023-02-14

**Authors:** Yujin Liu, Zhong Ji, Guobiao Cen, Hengchao Sun, Haibao Wang, Chuanxi Zhao, Zhong Lin Wang, Wenjie Mai

**Affiliations:** 1 Siyuan Laboratory, Guangzhou Key Laboratory of Vacuum Coating Technologies and New Energy Materials, Guangdong Provincial Key Laboratory of Optical Fiber Sensing and Communications, Guangdong Provincial Engineering Technology Research Center of Vacuum Coating Technologies and New Energy Materials, Department of Physics, Jinan University, Guangzhou, Guangdong 510632 China; 2 Guangzhou Institute of Technology, Xidian University, Guangzhou, Guangdong 510555 China; 3 Beijing Smart-Chip Microelectronics Technology Co., Ltd., Beijing, 100192 China; 4 CAS Center for Excellence in Nanoscience, Beijing Key Laboratory of Micro-Nano Energy and Sensor, Beijing Institute of Nanoenergy and Nanosystems, Chinese Academy of Sciences, Beijing, 100083 China; 5 School of Materials Science and Engineering, Georgia Institute of Technology, Atlanta, GA 30332 USA

**Keywords:** Optoelectronic devices and components, Imaging and sensing

## Abstract

There are two primary types of photoreceptor cells in the human eye: cone cells and rod cells that enable color vision and night vision, respectively. Herein, inspired by the function of human visual cells, we develop a high-resolution perovskite-based color camera using a set of narrowband red, green, blue, and broadband white perovskite photodetectors as imaging sensors. The narrowband red, green, and blue perovskite photodetectors with color perceptions mimic long-, medium-, and short-wavelength cones cells to achieve color imaging ability. Also, the broadband white perovskite photodetector with better detectivity mimics rod cells to improve weak-light imaging ability. Our perovskite-based camera, combined with predesigned pattern illumination and image reconstruction technology, is demonstrated with high-resolution color images (up to 256 × 256 pixels) in diffuse mode. This is far beyond previously reported advanced perovskite array image sensors that only work in monochrome transmission mode. This work shows a new approach to bio-inspired cameras and their great potential to strongly mimic the ability of the natural eye.

## Abstract (web summary)

We developed a human visual-cell-inspired perovskite-based color camera that demonstrated high-resolution (up to 256×256 pixels) color images in diffuse mode.

## Graphical abstract

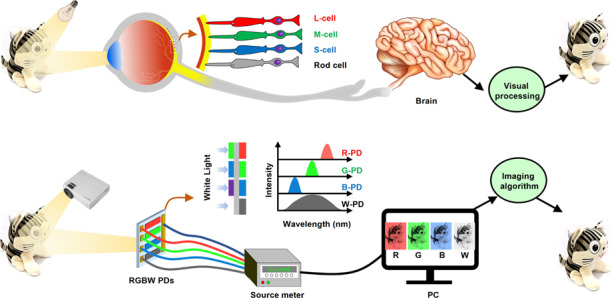

## Introduction

Human visual systems can perceive and respond to our surroundings and provide humans with the gifts of survival and learning. Intriguingly, thanks to the ability to learn from nature, humans have invented cameras that have drastically changed our lives. Today, commercial cameras have demonstrated a series of advantages of high resolution, fast imaging, and smart functionalities with the development of silicon-based charge-coupled devices (CCD) and complementary metal-oxide-semiconductor (CMOS) digital technology. However, there are still large gaps between advanced cameras (have developed over 100 years) and sophisticated biological eyes (have evolved over 100 million years)^1^. For instance, insect compound eyes exhibit a wide field of view and extreme position sensitivity, while mantis shrimp eyes^2^ combine the multispectral and polarization sensing. As another instance, the human eye^3^ has an adjustable pupil with light intensity, a lens with adjustable focus, a curved retina, as well as color vision cells.

Color vision is one of the key features of the human visual system. The reason why a human can enjoy this colorful world is due to the red (long wavelength, L), green (medium wavelength, M), and blue (short wavelength, S) cones in the retina^4^. In addition, rod cells, which have higher sensitivity than the cone cells, can help imaging under weak light. To realize the color vision, conventional imaging methods based on silicon-based photosensors usually adopt two approaches. One is to use dichroic prisms or optical gratings to split white light into S, M, L wavelengths, and the other way is to combine a set of red, green, and blue bandpass filters with three broadband photodetectors (PDs). Either way, the required optical components will increase the complexity of the imaging system. In addition, the inherent properties of silicon materials (e.g., poor mechanical properties and non-adjustable band gap) lead to their lack of competitiveness in mimicking biological vision systems.

Unlike crystalline silicon materials, orgnic-inorganic halide perovskite materials combine excellent optoelectronic properties (such as high light absorption coefficient, high mobility, and low binding energy) and flexibility in adjusting physical and chemical properties^5^, providing great potential for biomimetic optoelectronic devices. In terms of the visual system of the human eye, the ion migration properties of perovskites provide the physical basis for the bionic visual synapses^6,7^, and the tunable bandgap properties of perovskites offer the basis for the construction of bionic visual photoreceptors with color recognition^8–10^. Xue et al. reported a perovskite image array (10 × 10 pixels) with color imaging capability using a surface-charge recombination mechanism to construct narrowband CsPbX_3_ PDs^11^. Tsai et al. designed narrowband perovskite PD based on optical microcavities, realizing high-performance filterless artificial photoreceptors^12^. In addition, Gu et al. developed a biomimetic eyeball by using a hemispherical retina with a high-density perovskite nanowire, and demonstrated the basic human function of acquiring images^13^. Although this development greatly advanced the progress of bionic optoelectronics, the current imaging mode still uses the point-to-point array image sensors in the conventional imaging system. Therefore, most perovskite imaging systems suffer from low-resolution imaging due to the highly complex fabrication process. In addition, most of the above systems were only demonstrated with direct illumination in the transmission mode like laser scanners. In the strict sense, cameras or artificial eyes must operate in diffuse mode to capture much weaker diffuse light of a few orders of magnitude lower in intensity.

In recent decades, an algorithm-assisted single-detector imaging technique^14,15^ has been served as a promising alternative technique to conventional imaging by using a single detector and predesigned light patterns to replace detector arrays and bulky optical components. This imaging technique also provides an attractive platform that mimics a biological vision system for making simpler and cheaper but more powerful cameras^16,17^. Recently, we developed a novel field-of-view tunable wide-angle camera by combining a single large-area flexible perovskite PD and an advanced Fourier imaging algorithm^18^. The results demonstrated the great appeal of combining imaging algorithms with new optoelectronic devices.

Herein, a high-resolution color camera inspired by the human visual cell is developed by designing a set of red (R), green (G), and blue (B) self-filtering narrowband perovskite PDs mimicking L, M, S-cone cells and Fourier imaging algorithm. Compared with the traditional imaging system, which is a combination of CCD/CMOS and a set of lenses, our developed imaging system is simpler and cheaper using perovskite PD as an imaging device. The perovskite-based camera can achieve high-resolution images with 256×256 pixels in diffuse mode, far beyond the state-of-the-art perovskite-based artificial eyes^11,13,19^ or other perovskite imaging systems^20–22^. We further studied the color imaging features at bright illumination conditions and showed excellent color reproduction and high-resolution results at the same time. More importantly, in order to further endow the ability of imaging under weak light, we integrate a broadband white (W) perovskite PD (mimicking the rod cell) with better photodetection performance than narrowband PDs into the proposed imaging system. The added W PD can improve the imaging resolution of the perovskite-based camera under weak light. It is expected that a high-resolution color imaging system beyond the human eye in weak light can be obtained by further image fusion technology and higher-performance PDs.

## Results

**Fig. 1:**
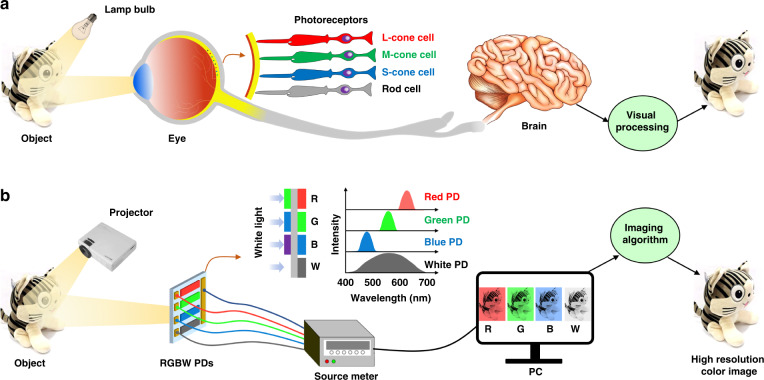
Human visual cell inspired perovskite-based color camera. **a** Schematic of the human visual system with L-, M-, S-cone and rod cells. **b** The proposed imaging system with four types of perovskite PDs (RGBW PDs) acting as L, M, S-cone, and rod cells (one-to-one correspondences with **a**) using a predesigned pattern illumination and image reconstruction technology

Figure 1 shows a comparison of the human visual system and our perovskite-based color camera (Fig. 1a, b). The human visual system mainly consists of two organs, the eye and the brain. The human eye has complex optical components (such as tunable lenses and an adjustable iris) and about 127 million photoreceptors covering the curved retina (about 7 million cone cells at macular areas of the center of the retina and about 120 million rod cells at retina area). This enables features of focal length tunability, light intensity modulation, high-resolution color imaging with low aberration, and highly sensitive night vision. At the same time, the human brain can process the neural signals from about a million optic nerve fibers in parallel, enabling high-speed visual processing and recognition. In a word, the human enjoyment of this colorful world is due to the presence of L, M, and S- cones in the retina and visual processing in the brain. In addition, as mentioned above, rod cells can help weak-light imaging.

Figure 1b illustrates the schematic of our perovskite-based color camera, which consists of a projector, four perovskite PDs (RGBW PDs), and a computer. The projector, which is connected to the computer, can process uniform illumination light into a series of predesigned structured light patterns that are produced by the four-step phase-shifting method^15^. The backscattered light intensity of each structured light pattern is measured by our fabricated perovskite PDs. The collected current values obtained by our PDs are further processed into high-quality images by the Fourier algorithm. More detailed information about the imaging system and algorithm can be found in Fig. S1 and Movie S1. Figure S1 illustrates the imaging principle. To better demonstrate the imaging process, we provided a video (Movie S1) that is a cartoon representation of the entire imaging process. Based on this method, we can overcome the difficulty of fabricating high-density array perovskite PDs. As shown in Fig. 1b, the narrowband R, G, B, and broadband W perovskite PDs mimic exactly the functions of the L-, M-, S- cones and rod cells, respectively. The narrowband RGB PDs are responsible for collecting information about red, green and blue colors in the imaging objects, which are used to create the final color image. The broadband W perovskite PD is used to realize the weak-light imaging ability that mimics the rod cells due to its better photodetection performance.

**Fig. 2:**
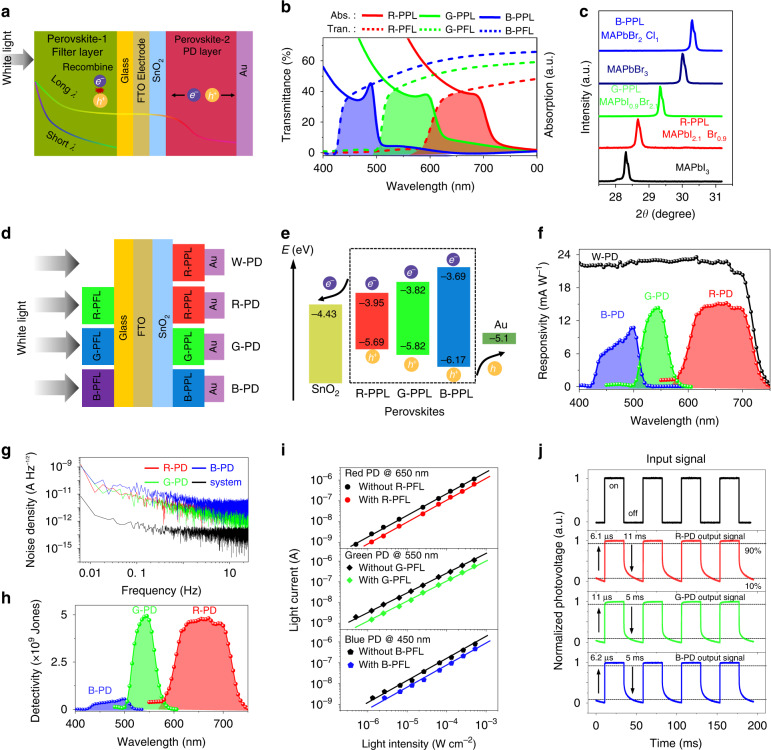
Perovskite-based RGBW PDs. **a** Schematic of the narrowband perovskite PDs with PFL and PPL (conceptual illustration). **b** Experimental transmittance spectra of PFLs and experimental absorption spectra of the PPLs. **c** Experimental XRD patterns on a narrow scale of the MAPbX_3_ (X = Cl, Br, I or mixed) perovskites for observing the trend of peak position change. **d** The device structure of the perovskite-based RGBW PDs (conceptual illustration). **e** Illustration of energy band of the RGB PPLs. **f** Experimental wavelength-dependent responsivity curves of the RGBW PDs. **g** Analysis of noise spectral density by Fourier transforming the experimental dark current waveform. **h** Wavelength-dependent specific detectivity curves obtained by equation D* = *R·A*^*1/2*^*/i*_*n*_. **i** Light currents as a function of light intensity. **j** Transient photovoltage curves of the RGBD PDs

Spectrally selective narrowband photodetection is essential for color or multispectral imaging^11,12,23^. Traditional color detection is carried out using optical bandpass filters or light-splitting elements such as optical prisms and gratings. However, this approach increases the complexity of the architecture and limits the quality of color sense. Metal halide perovskites, as a class of emerging low-cost, solution-processable semiconductor materials with tunable optoelectronic properties, bring new solutions for high-performance narrowband PDs^9,10^. Importantly, the bandgap of metal halide perovskites can be easily adjusted by changing the composition of the halogen element (I, Br, Cl). In this work, we adopted a new strategy of self-filtering narrowband perovskite PDs as shown in Fig. 2a. The main idea of this strategy is to introduce a perovskite filter layer with shorter wavelength absorption against PD perovskite with longer wavelength. As illustrated in Fig. 2a, when the white light illuminates, the shorter wavelength is absorbed by the perovskite filter layer (PFL), but does not generate photocurrent. Also, the longer wavelength is absorbed by the perovskite PD layer (PPL) after passing through the PFL, generating photocarriers to form photocurrent. According to this strategy, the narrowband response of a specific wavelength can be designed by choosing two perovskite layers with different bandgaps. Therefore, red, green, blue perovskite filter layers (R-PFL, G-PFL, B-PFL), and red, green, blue perovskite PD layers (R-PPL, G-PPL, B-PPL) were designed by a series of bandgap engineering (Fig. S2). The matching of the appropriate PFL and PPL will generate the narrowband RGB PDs. Figure 2b shows the transmittance spectra of the perovskite filter layers and the absorption spectra of the perovskite PD layers. The overlapped wavelengths between X-PPL and X-PFL (X is R, G, B) indicate their specific narrowband response of final PDs. All of the absorption spectra of the 6 perovskites (3 PFLs, 3 PPLs) are shown in Fig. S3. We further analyzed the X-ray diffraction (XRD) patterns of MAPbX_3_ perovskites (R, G, B-PPLs) and the results are shown in Fig. 2c. The results indicate that the introduction of Br/Cl ions into MAPbI_3_/MAPbBr_3_ causes a systematic shift of the (200) peak towards a higher 2*θ* range of 28–31°, which is consistent with previous reports^8^. The XRD spectra of these MAPbX_3_ perovskites are shown in Fig. S4. After the preparation of the above perovskite PFL and PPL, we integrated the perovskite-based RGBW PDs on a glass substrate. The results are shown in Fig. 2d. It is worth noting that the W PD consists of an R PPL (MAPbI_2.1_Br_0.9_) without an R-PFL and thus has a wider spectral response and better responsiveness. The spin-coated SnO_2_ film was used as electron transport layers (ETL) to improve the performance of the color perovskite PDs. The picture of the color PDs is placed in Fig. S5, and the cross-sectional SEM image of the PPL is shown in Fig. S6. Figure 2e presents the energy-level diagram of the color perovskite PDs. The band energy levels of the RGB PPL were calculated using absorption spectra and ultraviolet photoelectron spectroscopy (UPS) spectra and are shown in Fig. S7. SnO_2_ as the ETL can block the holes and transport electrons, enabling the reduction of photo-carriers recombination. Therefore, the matched band alignment at the color perovskites/SnO_2_ interface provides an excellent photodetection performance for narrowband perovskite PDs.

Figure 2f shows the narrowband response curves of the perovskite-based narrowband RGB PDs and the broadband response curve of the perovskite W PD. It indicates that our RGB perovskite PDs exhibit satisfactory narrowband response and decent responsivity in short- (6.5 mA W^−1^ at 450 nm), middle- (13.7 mA W^−1^ at 550 nm), long-wavelength (15 mA W^−1^ at 650 nm), and perfectly mimic the color S-, M-, L- cones of human eyes. Moreover, the W PD mimicking rod photoreceptor shows better photodetection performance (responsivity of 22.6 mA W^−1^ at 650 nm) than the RGB PDs, implying better detection ability under weak light. The responsivity of these perovskite PDs is measured at zero bias due to the built-in electric field at the SnO_2_/perovskite film interface. The self-powered ability of our RGBW perovskite PDs helps further simplify our color camera. As shown in Fig. 2g, we further analyzed the noise spectral density of the RGB PDs by Fourier transform of the time–domain waveforms of dark currents. In summary, all the RGB PDs show low noise levels, and the B-PD presents more noise fluctuations (noise level of 3 × 10^–12^ at 1 Hz bandwidth) than the R- (noise level of 1 × 10^–12 ^A Hz^−1/2^ at 1 Hz bandwidth) and G-PDs (noise level of 9 × 10^–13 ^A Hz^−1/2^ at 1 Hz bandwidth), indicating weaker detection performance. The specific detectivity at different wavelengths of the RGB PDs further confirms the above results as shown in Fig. 2h. Interestingly, this result is coincidentally consistent with the characteristics of the human eye, that is, the blue cone cells are less sensitive than the red and green cones. Figure 2i shows the excellent linear response characteristics of our perovskite PDs. The responsivity at different light intensities of these PDs is shown in Fig. S8. The response speed of imaging PDs is an important parameter to limit the speed of the imaging system. Figure 2j presents the transient photoresponse curves. We calculated the rise/decay time by fitting the rising and falling edges, and obtained the corresponding response time, as shown in Fig. S9. The results indicate that our PDs exhibit fast microsecond response times compared with the reported PDs^24–28^. In short, we have prepared a set of appropriate perovskite PDs that mimic human cone cells and rod cells, with good performance (comparisons with other perovskite photoreceptors are shown in Table S1) for the perovskite-based color imaging system.

**Fig. 3:**
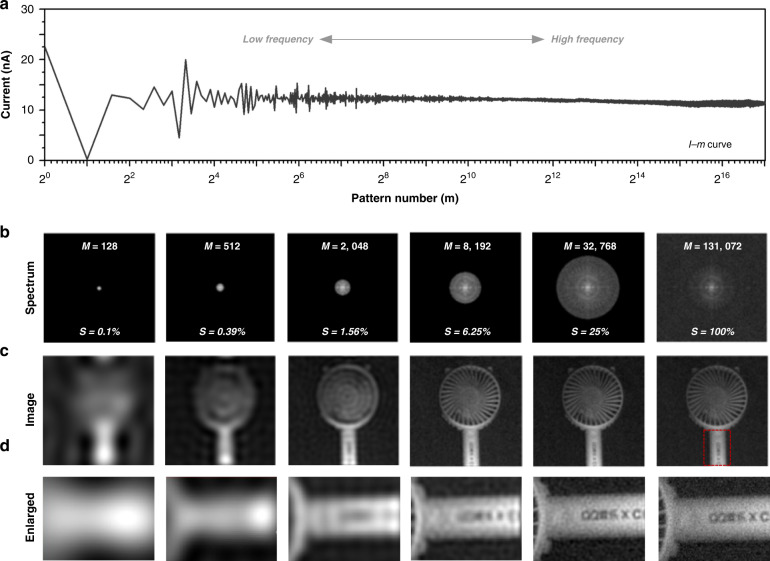
Demonstration of high-resolution imaging with 256 × 256-pixels. **a** Obtained *I-m* curve (*M* = 1: 131, 072, and 1 point per pattern) by the perovskite W PD. **b** Fourier coefficient spectrum (logarithmic modulus) when the measurement number (*M*) increases (from left to right). **c** The corresponding reconstructed image. **d** The enlarged image

To demonstrate the high-resolution imaging capability of our perovskite PD-based imaging system, the high-resolution imaging results obtained by the perovskites W PD are shown in Fig. 3. The curve in Fig. 3a is the current-projected pattern number (*I-M*) curve, from which it can be seen that the acquired current intensity changes with the change of the projected pattern. In particular, the current intensity difference within a step-four phase shift pattern *[D*_*0*_*(f*_*x*_*,f*_*y*_*) − D*_*π*_*(f*_*x*_*,f*_*y*_*) or D*_*π/2*_*(f*_*x*_*,f*_*y*_*) − D*_*3π/2*_*(f*_*x*_*,f*_*y*_*)]* whether it is low frequency or high frequency, has a very high signal-to-noise ratio, as shown in the related figures. So, the Fourier coefficients calculated based on the differences (*α*_*Re*_ = *D*_*0*_*(f*_*x*_*,f*_*y*_*) - D*_*π*_*(f*_*x*_*,f*_*y*_*)* and *α*_*im*_ = *j·[D*_*π/2*_*(f*_*x*_*,f*_*y*_*) − D*_*3π/2*_*(f*_*x*_*,f*_*y*_*)])* are reliable. As is well known, a 2D image can be converted to the frequency domain by Fourier transform. In the Fourier spectrum, the low-frequency information determines the general outline of the imaging target, and the high-frequency information determines the details. Therefore, with full sampling, the reconstructed image has a good outline and yet many details, which means high resolution. However, with less sampling, the resolution decreases. Here, to descript the degree of under-sampling, we define a new factor as: spectrum coverage (*S*) = number of obtained Fourier coefficients/ number of required Fourier coefficients

Here, full sampling of an image requires *M* = 2 × *I* × *J* measurements, where *I* and *J* are the horizontal and vertical pixels of the image. For instance, a full sampling of a 256 × 256 image requires *M* = 131,072 measurements. Figure 3b shows the obtained Fourier spectrum (logarithmic modulus) when the measurement number (*M*) increases (*S* increases) and the corresponding reconstructed image (Fig. 3c, d). With full sampling (*M* = 131,072), the reconstructed image is well outlined yet detailed, that is, it has high resolution. However, with fewer patterns, the resolution decreases. The results show that the resolution can be easily improved by increasing the measurement number (*M*), and in particular, images with dimensions 256 × 256 pixels can be achieved by adopting *M* > 131,072 measurements.

**Fig. 4:**
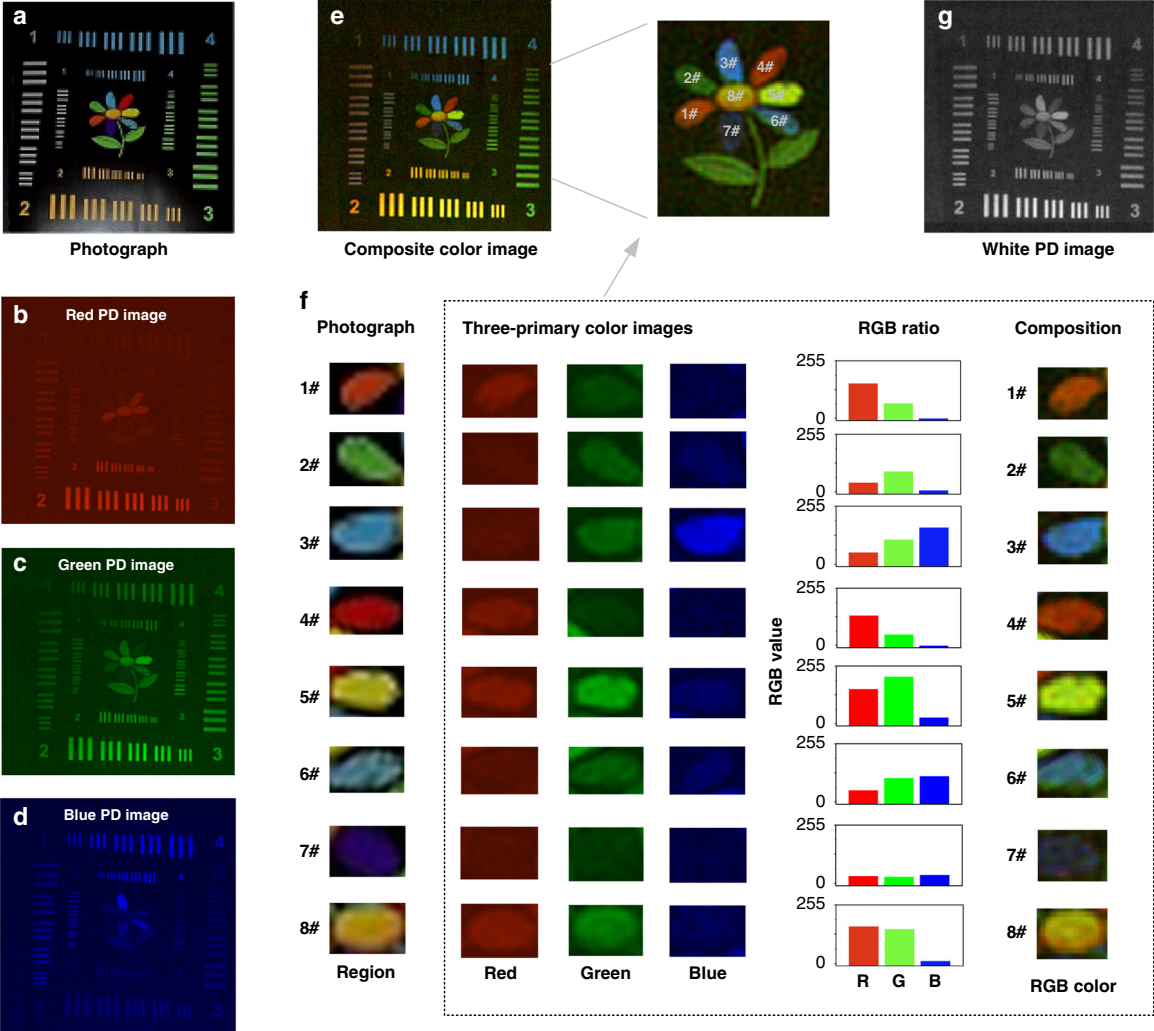
Demonstration of color imaging under a diffuse light intensity of ~20 μW cm^−2^. **a** Photograph of the imaging target. **b**–**d** Images by RGB PDs. **e** Composite color image of RGB PD images. Inset is the enlarged color flower petals marked by numbers. **f** The comparison of each flower petals obtained by photograph, individual RGB images, and the final composed color image. **g** Image by W PD

The color imaging can be realized by using the narrowband RGB perovskite PDs to record the current value simultaneously and using a subsequent reconstruction algorithm. The photograph of the imaging target is shown in Fig. 4a. The monocolor imaging results by the RGB PDs are obtained and shown in Fig. 4b–d. By combining the information obtained from the RGB images and using the black and white areas for normalization and balance^29^, the color image is composed as shown in Fig. 4e, and the detailed composition process is shown in Fig. 4f. Figure 4f shows that the target image is successfully composed by combining the RGB images, which means that the proposed imaging system has excellent color reproduction. The slight differences in #5 and #7 are mainly due to different illumination light sources. Here, in this paper, the light intensity refers to the diffuse light in the position of the PDs and is 20 μW cm^−2^ in this condition. Therefore, it can be concluded that the composite color image has similar good quality when the illumination light is strong (>20 μW cm^−2^), just as the human eye is good at seeing colored objects with sufficient light intensity. In addition, because the four PDs have an inevitable slight position difference during the imaging progress, we also investigated the influence of the PD position on the imaging results. The results are shown in Fig. S10. The results show that the small differences in the position of the PDs do not change the field of view, which is mainly determined by the projection area.

**Fig. 5:**
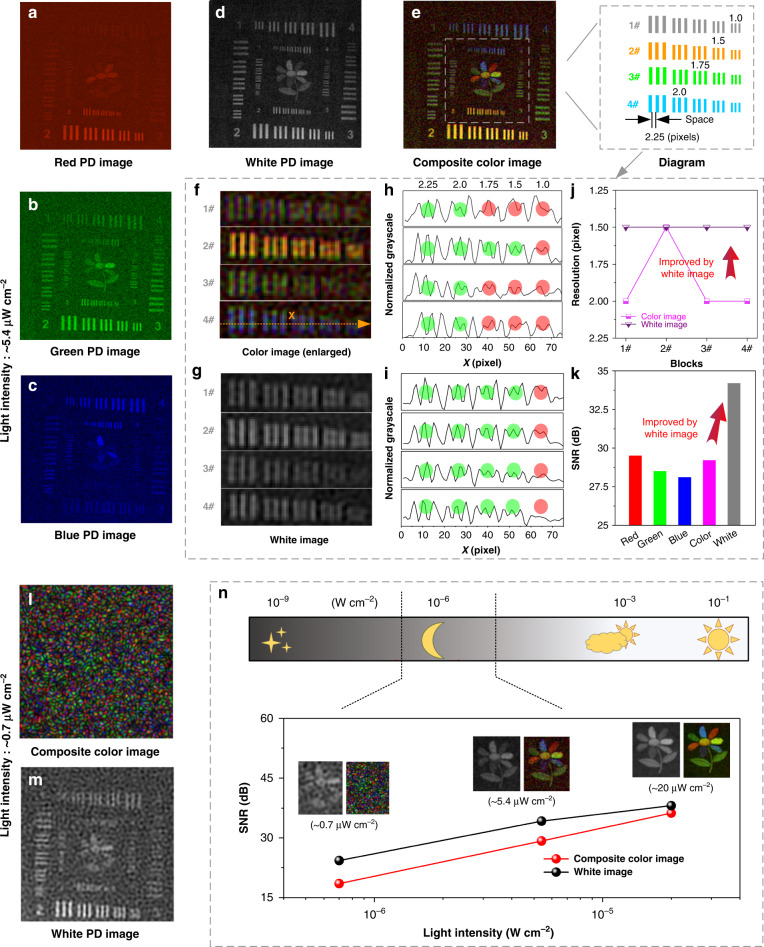
Demonstration of color imaging with weaker light illuminations. **a**–**d** Images by narrowband RGB PDs and broadband W PD under a light intensity of ~5.4 μW cm^−2^. **e** Composite color image. Inset is a diagram of space between blocks for calculating resolution. **f**, **g** Enlarged images of blocks are depicted in the inset of **e**, respectively. **h**, **i** Normalized grayscale of line x in **f**, **g**, respectively. **j** Calculated resolution. **k** Calculated signal-to-noise ratio (SNR) of images. **l**, **m** Composite color and white images were obtained by our camera under a light intensity of ~0.7 μW cm^−2^. **n** A summary exhibition of imaging results of our perovskite-based color camera under different light intensities

When the light intensity becomes weaker to ~5.4 μW cm^−2^, the resolution and signal-to-noise ratio (SNR) of the reconstructed color image deteriorate, as shown in Fig. 5e, due to the worse RGB PD images (Fig. 5a–c). However, as shown in Fig. 5d, the W PD perovskite image has higher resolution and SNR because of its higher response and wider response spectrum than the RGB PD perovskite. Therefore, the white PD can assist the color image to get a better image quality by color/white image fusion using a simple image overlay method. This is similar to human rod cells that can help color imaging under weak light (Fig. S11). Quantitatively, in the magnified images (Fig. 5f, g), a line (line x) can be drawn to show the overlapping curves where two lines are close together to calculate resolution. According to the Rayleigh criterion, the smallest distinguishable distance is resolution, and the corresponding results are shown in Fig. 5h, i. Here, the unit of resolution is a pixel and we can easily convert it to distance by multiplying separable pixels by the distance between pixels. The calculated results show that the resolution of the white image is better than the composite color images, as shown in Fig. 5j. Furthermore, as shown in Fig. 5k, the SNR of the white image is also better (note that the SNR is defined as SNR = 20 × log [Signal/Noise] where Signal is the mean intensity of the signal area and Noise is the variance of the noise area). In a word, the W PD simulating rod cells play a role in improving the ability of color imaging in this situation. In the future, an artificial intelligence method instead of a simple image overlay method may be a better way for image fusion.

When the illumination light continually becomes even weaker (~0.7 μW cm^−2^), the light intensity is lower than the minimum detectable light intensity of RGB PDs (Fig. 2i). So, the RGB PDs cannot work properly. But the W PD shows a minimum detectable light intensity of 0.3 μW cm^−2^, so it can still work at a light intensity of 0.7 μW cm^−2^. The color image and white image obtained by the RGB PDs and white PD are shown in Fig. 5l, m. Obviously, in this low light, the proposed imaging system can no longer perform color imaging, but it can still achieve outline imaging of objects. Just like under the moonlight, rod cells help people see the outline of objects. Of course, extremely weak light («0.7 μW cm^−2^) may also blind the W PD, and therefore improving the specific detectivity of perovskite PDs is still an important research direction.

As a summary of Figs. 4–5, the W PD simulating rod cells may perform differently in the proposed imaging system when the light conditions are different. As shown in Fig. 5n, the SNR of white PD images is always higher than that of composite color images (24.3 dB *vs*. 18.5 dB at ~0.7 μW cm^−2^, 34.2 dB vs. 29.2 dB at ~5.4 μW cm^−2^, and 38.1 dB vs. 36.2 dB at ~20 μW cm^−2^), due to the better performance of the photodetection. When the light intensity is high enough (>20 μW cm^−2^), the RGB PD can provide high-quality color image with a wide spectral region. But when the light intensity turns weaker (~5.4 μW cm^−2^), the W PD contributes to the improvement of color imaging ability. If the illumination light becomes even weaker (~0.7 μW cm^−2^), the W PD helps see the outline of objects.

## Discussion

In this work, we have demonstrated a perovskite-based color camera based on the RGBW perovskite PDs to mimic the SML cone cells and rod cells of the human eye, combined with a predesigned pattern illumination and image reconstruction technology. With the proper performance of narrowband PDs, our camera can achieve color imaging under the weak light of ~5.4 μW cm^−2^. In addition, we have integrated a broadband perovskite W PD into our color camera to enhance the imaging capability in low-light environments, which can achieve the outline of imaging objects under the low light of ~0.7 μW cm^−2^. This work is believed to have opened a new horizon in the development of cameras and found a new path for mankind to imitate the natural eye.

## Materials and methods

### Preparation and characterization of the RGBW perovskite PDs

The self-filtering narrowband strategy benefits from the tunable bandgap process of the MAPbX_3_ perovskites. The narrowband perovskite photodetector (PD) consists of a perovskite filter layer (PFL) and a perovskite PD layer (PPL). Therefore, the fabrication of narrowband RGB PDs needs three PFLs and three PPLs. The fabrication process of these PFLs and PPLs are shown in Fig. S2. In order to better suppress the absorption of PPL at a short wavelength, the 2 M perovskite precursor solution was used to deposit PFLs, and the 1 M perovskite precursor solution was used to deposit PPLs. The entire preparation route of RGB PFLs and PPLs is shown in Fig. S2, where the spinning speed is 4000 rpm and the annealing temperature is 110 °C. The SnO_2_ film as the electron transport layer was pre-deposited on the substrate by the spin-coating method to improve the photo-detection performance of the perovskite PD device. An 80-nm Au electrode was deposited by thermal evaporation on the RGB PPL to form a SnO_2_/perovskite heterojunction PD device. The active area of the PD device is 0.1 cm^2^. The narrowband RGB perovskite PDs is combined with the corresponding RGB PFLs to make the narrowband RGB perovskite PD devices (short for RGB perovskite PDs). In order to use the device for a long time, we pack the devices to prevent rapid rotting of the device. The XRD patterns of as-prepared perovskite films were measured by X-ray diffractometer (Rigaku, MiniFlex600). The absorption spectra were measured by an ultraviolet–visible spectrum photometer (Shimadzu ultraviolet-2600). The valence-band alignments were measured by ultraviolet photoelectron spectroscopy UPS (bias of 1.5 V, He I radiation, hυ = 21.22 eV).

### Measurement of perovskite PDs

The photoresponse curves were measured by a source meter (2601B, Keithley, USA) under illumination of monochrome light (TLS3-X500A, Zolix, China). The output intensity can be adjusted by neutral optical attenuation plates. The spectral response (300–800 nm) curve of the PDs was measured using a QE-R external quantum efficiency instrument (Si detector S10-14010, Enlitech), and the photocurrent was recorded by the source meter (2601B, Keithley, USA). The transient photoresponse behaviors of PDs were measured by a home-built measurement system, using a photo-induced open-circuit voltage decay method with a high-speed digital oscilloscope and a corresponding pulsed laser.

### Imaging system and process

The schematic diagram of the experimental setup is shown in Fig. 1 and Fig. S1. Figure S1 (a) shows the detailed experimental setup of our color imaging system, which consists of a projector, four perovskite PDs (RGBW), and a computer. Figure S1 (b) presents the schematic diagram of the imaging algorithm. First, we use RGBW PDs to record a set of current intensities corresponding to several four-step phase-shifting sinusoidal patterns (*P*_*Φ*_(*f*_*x*_, *f*_*y*_), where *f*_x_ and *f*_y_ are 2D spatial frequency. As shown in Fig. S1 (b), the set of current intensities can be called the *I-m* curve, where *m* is the pattern number. Then, the *I-m* curves can be grouped into *D1-D4 (D*_*0*_*(f*_*x*_*,f*_*y*_*)* to *D*_*3π/2*_*(f*_*x*_*,f*_*y*_*))* depending on the four-step phase-shifting. Subsequently, the Fourier coefficients can be calculated using *α*_*Re*_ = *D*_*0*_*(f*_*x*_*,f*_*y*_*)* *−* *D*_*π*_*(f*_*x*_*,f*_*y*_*)* and *α*_*im*_ = *j·[D*_*π/2*_*(f*_*x*_*,f*_*y*_*)* *−* *D*_*3π/2*_*(f*_*x*_*,f*_*y*_*)]}*, where *α*_*Re*_ is the real part and *α*_*im*_ is the imaginary part. After that, the spatial reflectivity at a particular wavelength of imaging sample *R*_*i*_(*x*, *y*) in the matrix space can be restored by inverse Fourier transform *R*_*i*_*(x,y)* = *1/(2kRb)·Fft*^*−1*^*{α*_*Re*_ + *α*_*im*_*}*, where *Fft*^−1^ denotes the inverse Fourier transform operator, *R* is the responsibility, and *b* is the average contrast. As a result, monocolor images (R, G, B, and W) are acquired. Here, using the trichromatic method, the result of the composition is a color image, and then a high-resolution color image can be obtained by color/white fusion.

## Supplementary information

Supplementary Information for Perovskite-based color camera inspired by human visual cells

Perovskite-based color camera inspired by human visual cells
