# Supplementary material for: Perovskite-based color camera inspired by human visual cells
Source: Light Sci Appl. 2023 Feb 14;12:43. doi: 10.1038/s41377-023-01072-y (PMC9929324; doi:10.1038/s41377-023-01072-y)
Supplement: Supplementary file 1 — Supplementary Information for Perovskite-based color camera inspired by human visual cells [file 41377_2023_1072_MOESM1_ESM.docx]

**Supplementary Information for**

**Perovskite-based color camera inspired by human visual cells**

Yujin Liu^1,2#^, Zhong Ji ^1,2#^, Guobiao Cen^1^, Hengchao Sun^5^, Haibao Wang^5^, Chuanxi Zhao^1^, Zhong Lin Wang^3,4*^, Wenjie Mai^1,3*^

^1^ Siyuan Laboratory, Guangzhou Key Laboratory of Vacuum Coating Technologies and New Energy Materials，Guangdong Provincial Key Laboratory of Optical Fiber Sensing and Communications，Guangdong Provincial Engineering Technology Research Center of Vacuum Coating Technologies and New Energy Materials, Department of Physics, Jinan University, Guangzhou, Guangdong 510632, China.

^2^ Guangzhou Institute of Technology, Xidian University, Guangzhou, Guangdong 510555, China

^3^ CAS Center for Excellence in Nanoscience, Beijing Key Laboratory of Micro-Nano Energy and Sensor, Beijing Institute of Nanoenergy and Nanosystems, Chinese Academy of Sciences, Beijing 100083, China.

^4^ School of Materials Science and Engineering, Georgia Institute of Technology, Atlanta, GA 30332, USA.

^5^ Beijing Smart-Chip Microelectronics Technology Co., Ltd., Beijing, 100192, China

*Corresponding authors: [zhong.wang@mse.gatech.edu](mailto:zhong.wang@mse.gatech.edu)(ZLW);[wenjiemai@email.jnu.edu.cn](mailto:wenjiemai@email.jnu.edu.cn) (WJM)

# These two authors contributed equally to this work.


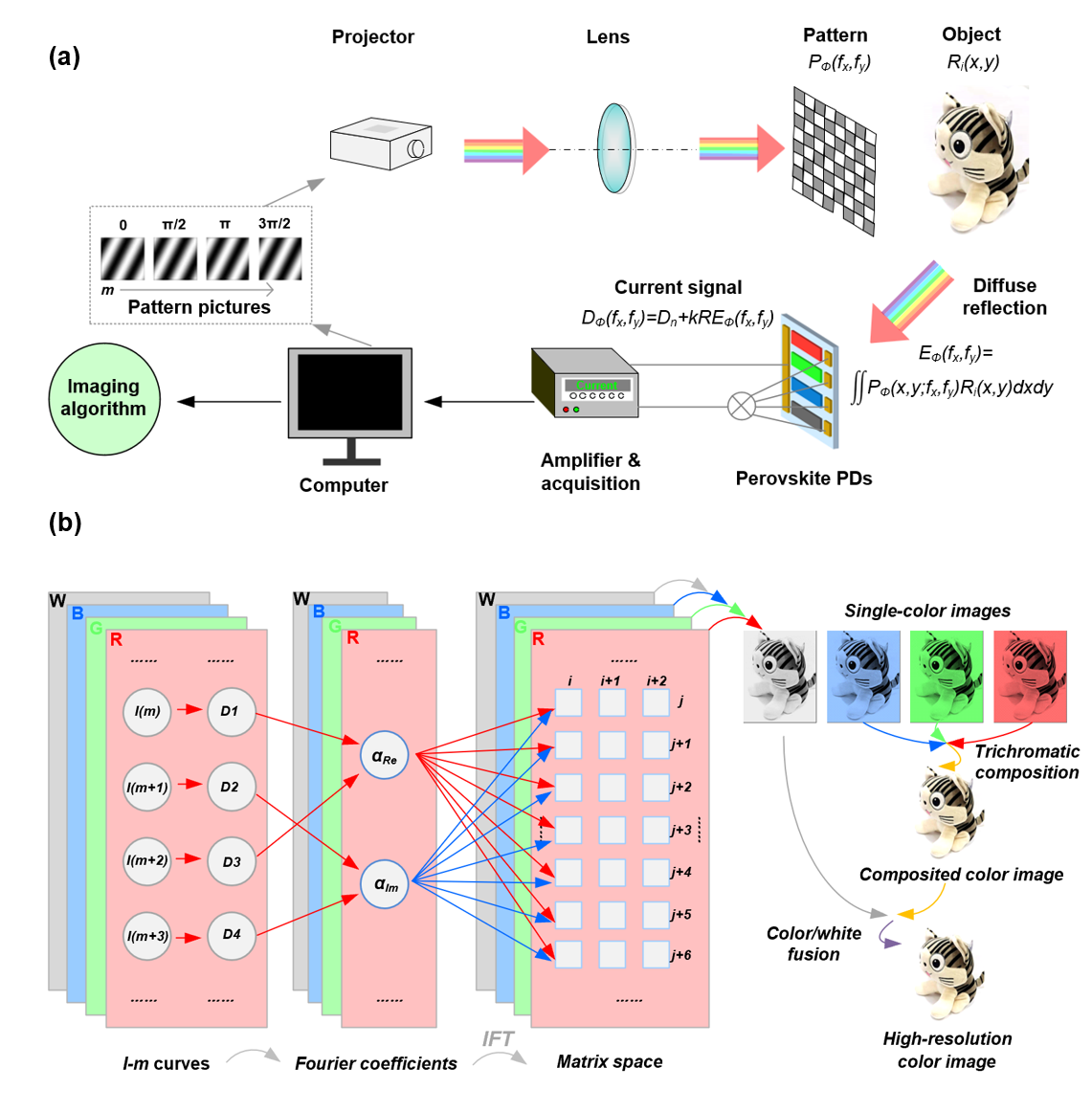


**Fig. S1.** (a) Detailed experimental setup of the Fourier single-pixel imaging system. (b) Schematic diagram of imaging algorithm.


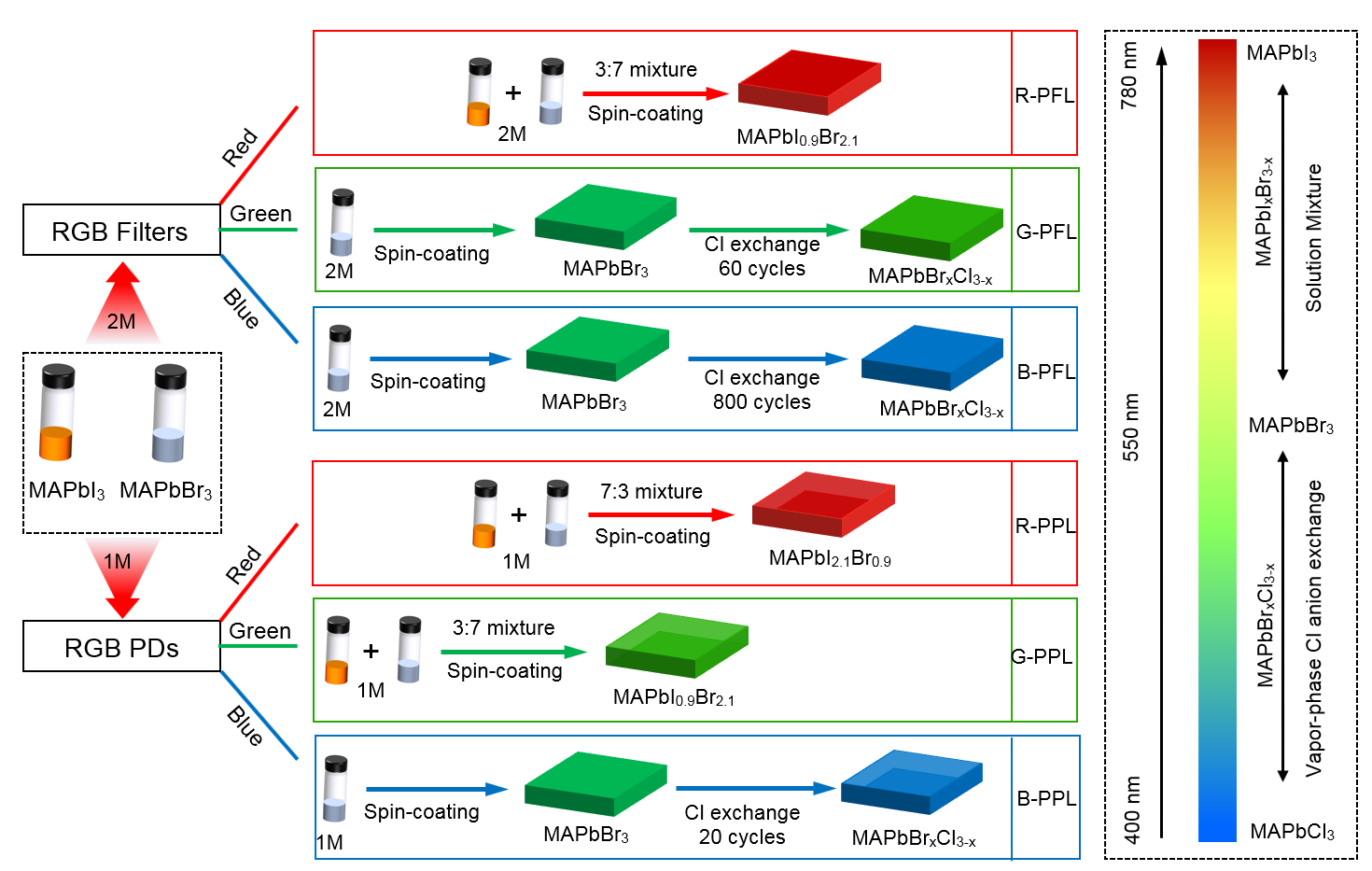


**Fig. S2.** Fabrication process of all of PFLs and PPLs.


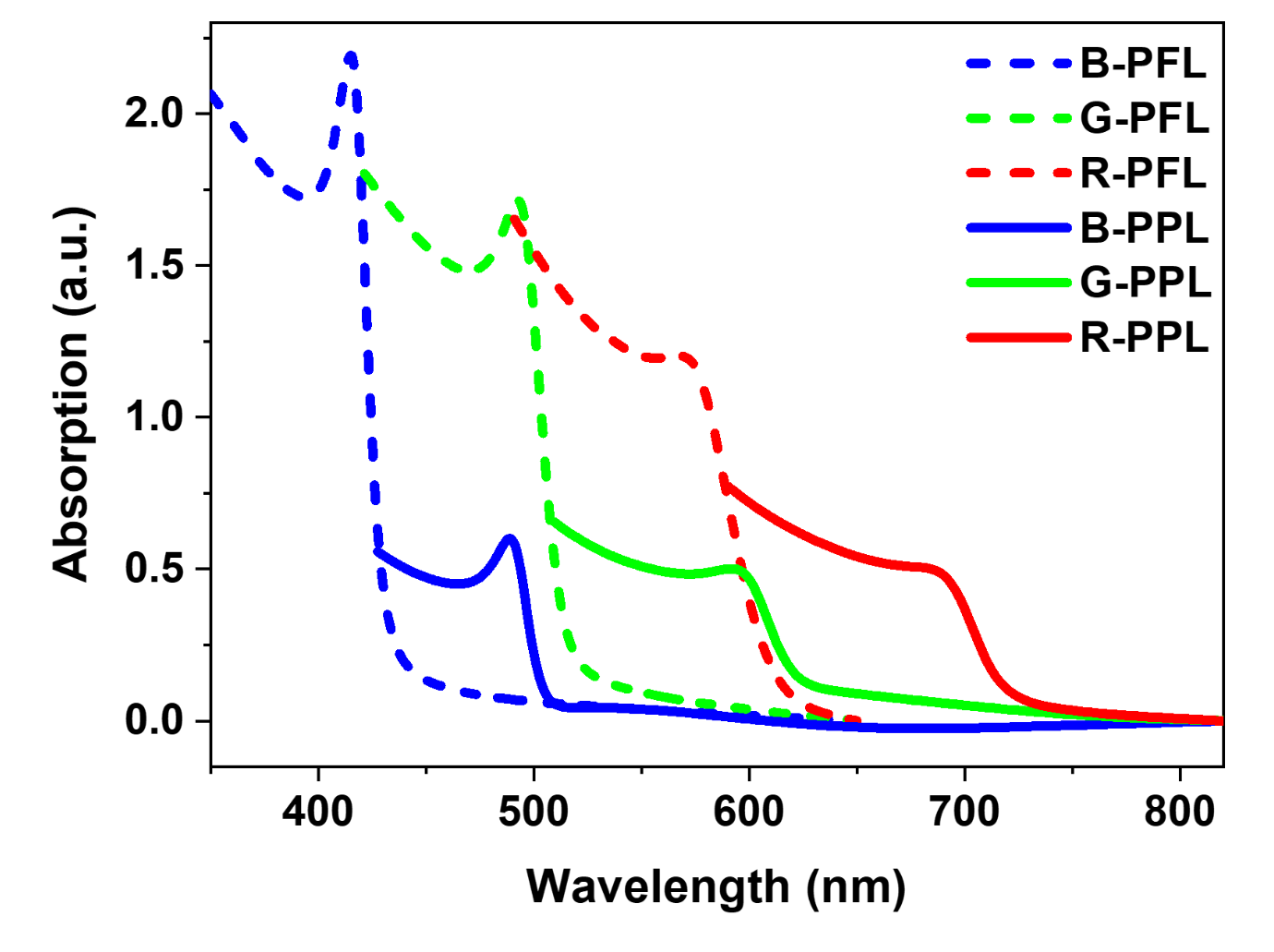


**Fig. S3.** All of absorption spectra of 6 perovskite films (3 PFLs, 3 PPLs).


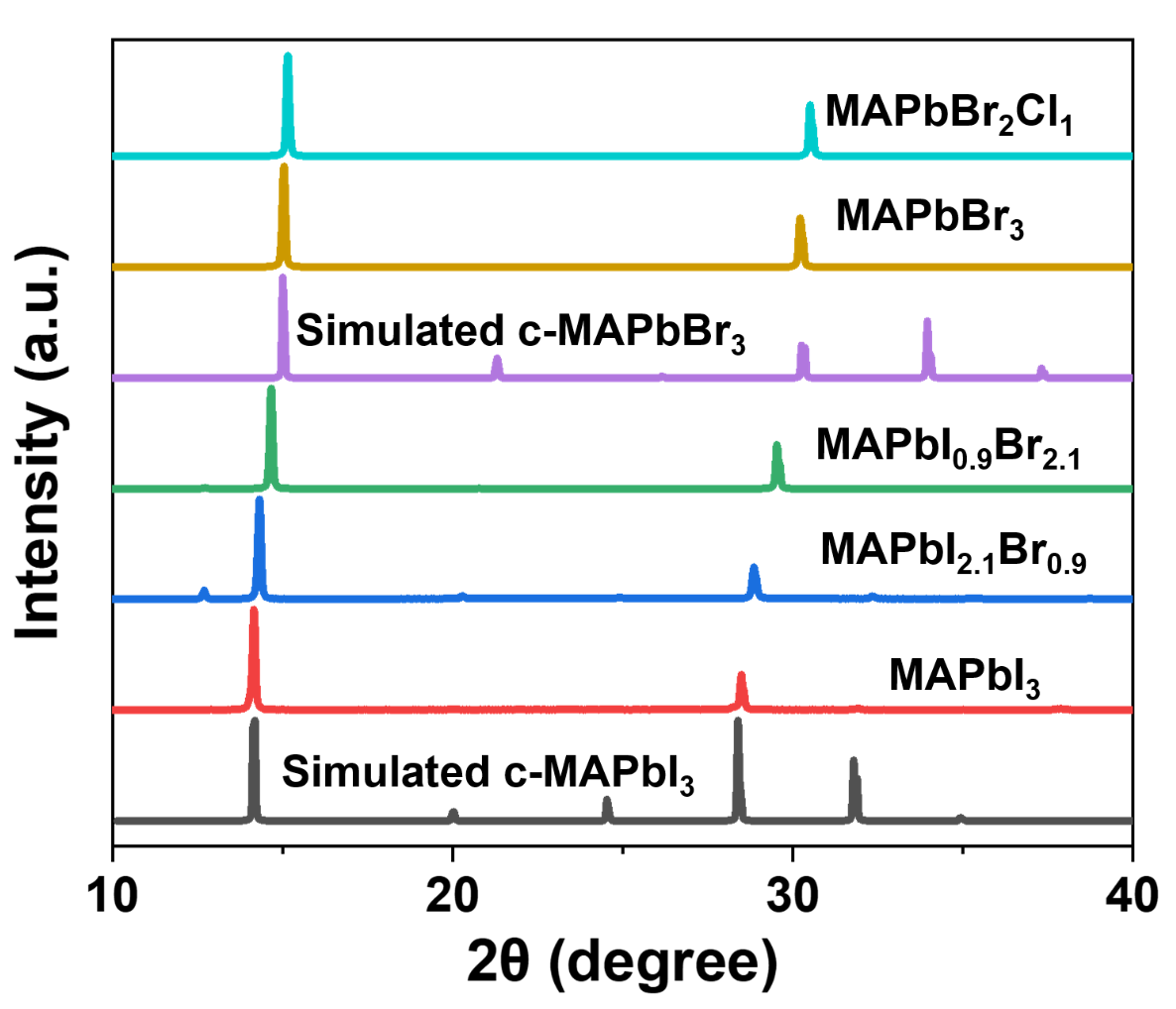


**Fig. S4.** The survey XRD spectra of these PPLs (MAPbX_3_).


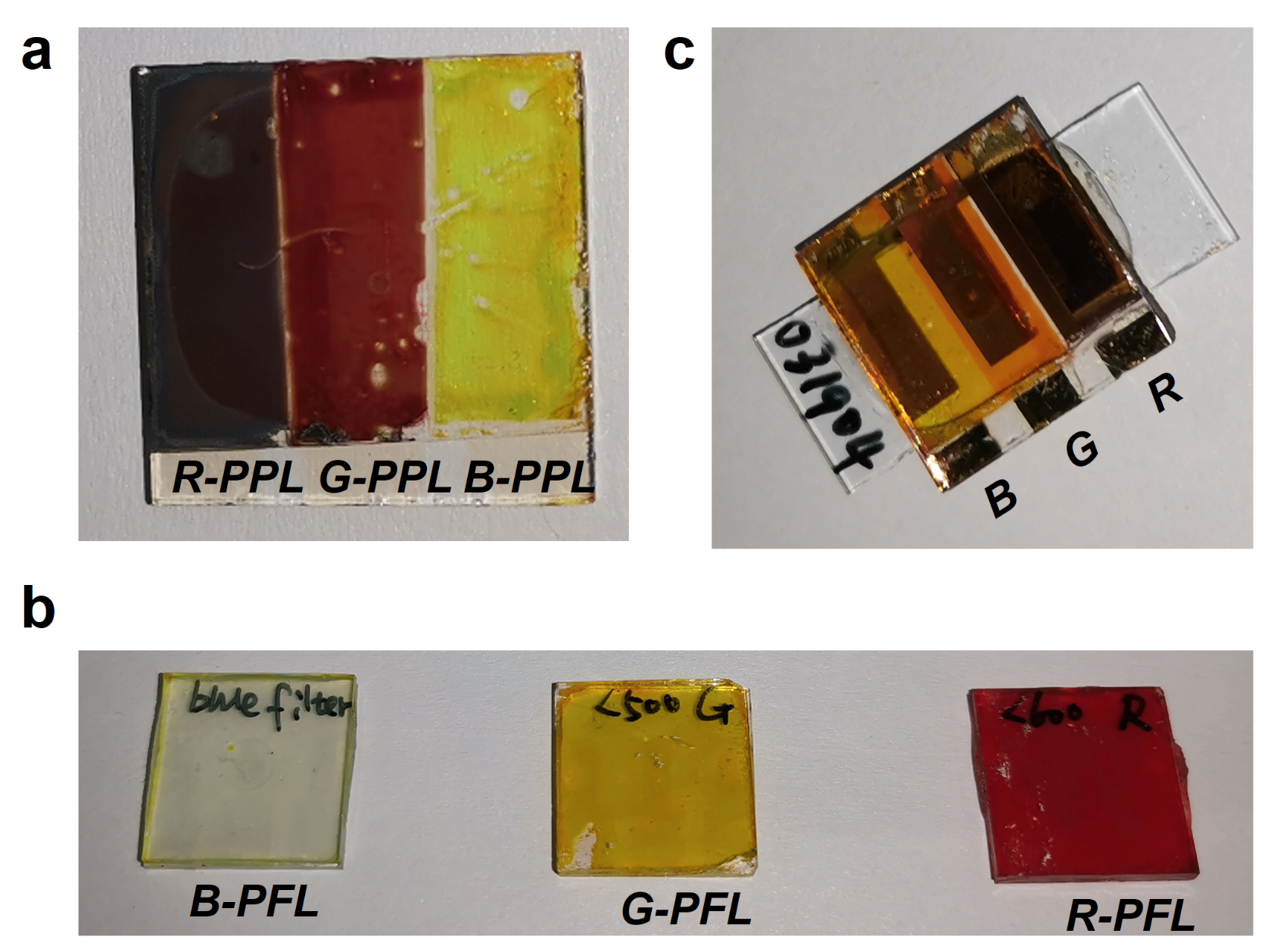


**Fig. S5.** The photographs of the PPLs (a), PFLs (b), and RGB PDs (c).


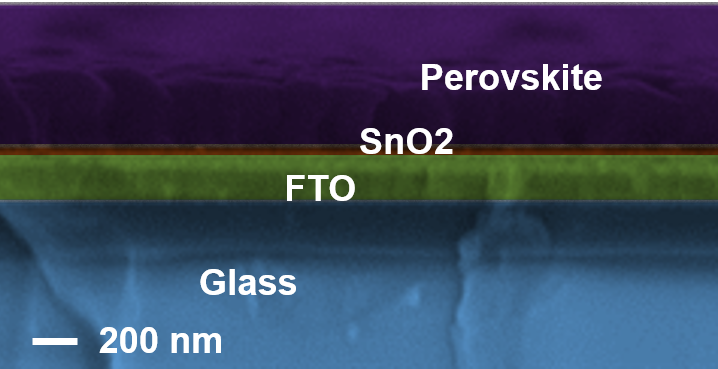


**Fig. S6.** The cross-sectional SEM image of the PPL.


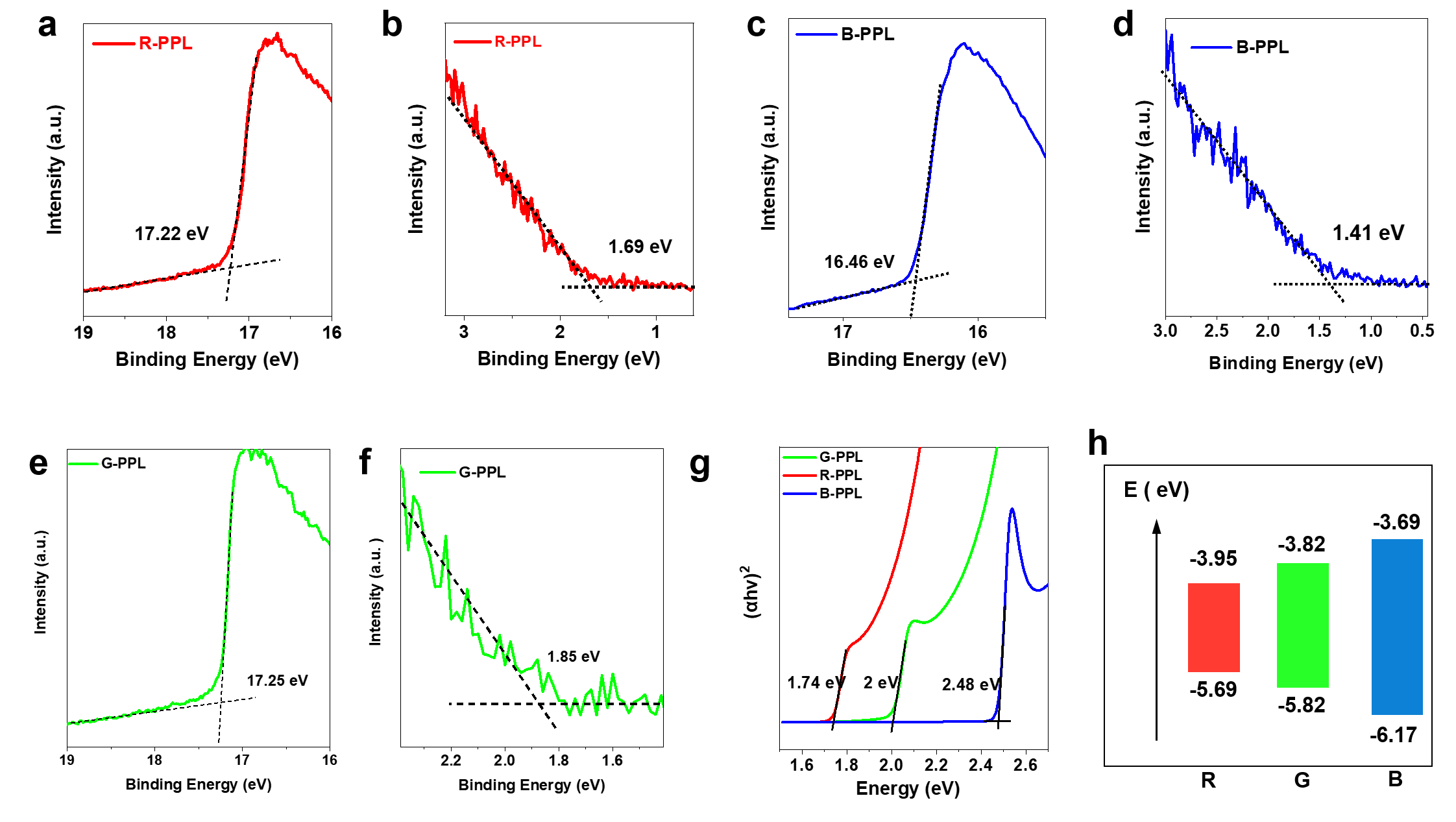


**Fig. S7.** The band-energy calculation of MAPbX_3_ perovskite films. (a-f) UPS spectra of RGB PPLs. (g) Absorption spectra of RGB PPLs. (h) Schematic diagram of the energy band alignment of RGB PPLs.


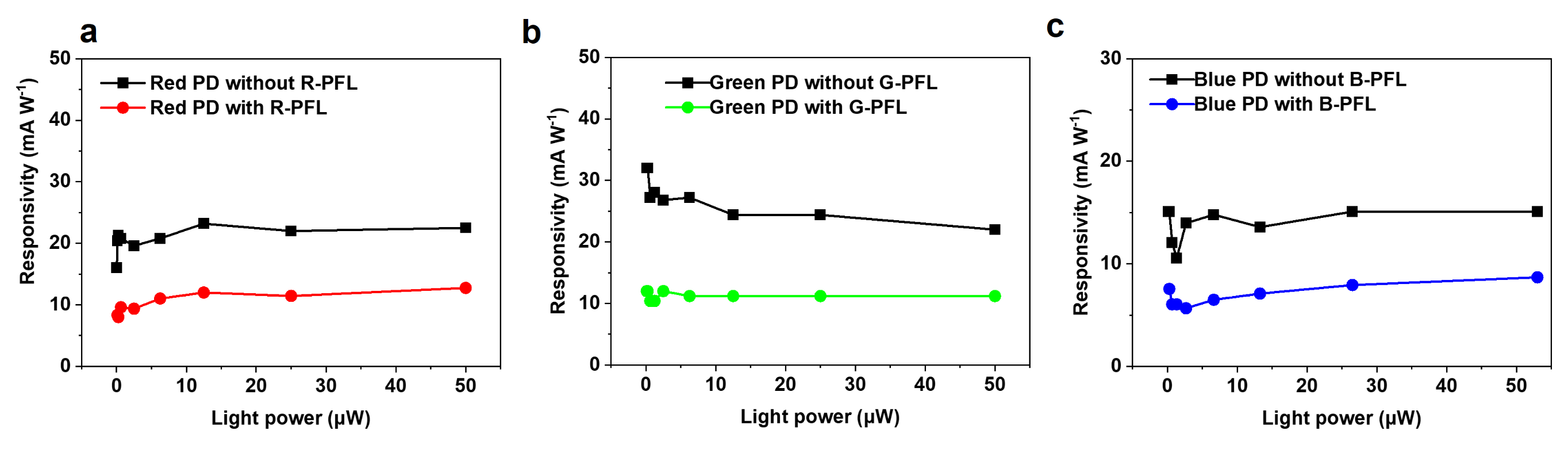


**Fig. S8.** The responsivity at different light intensity of narrowband RGB PDs


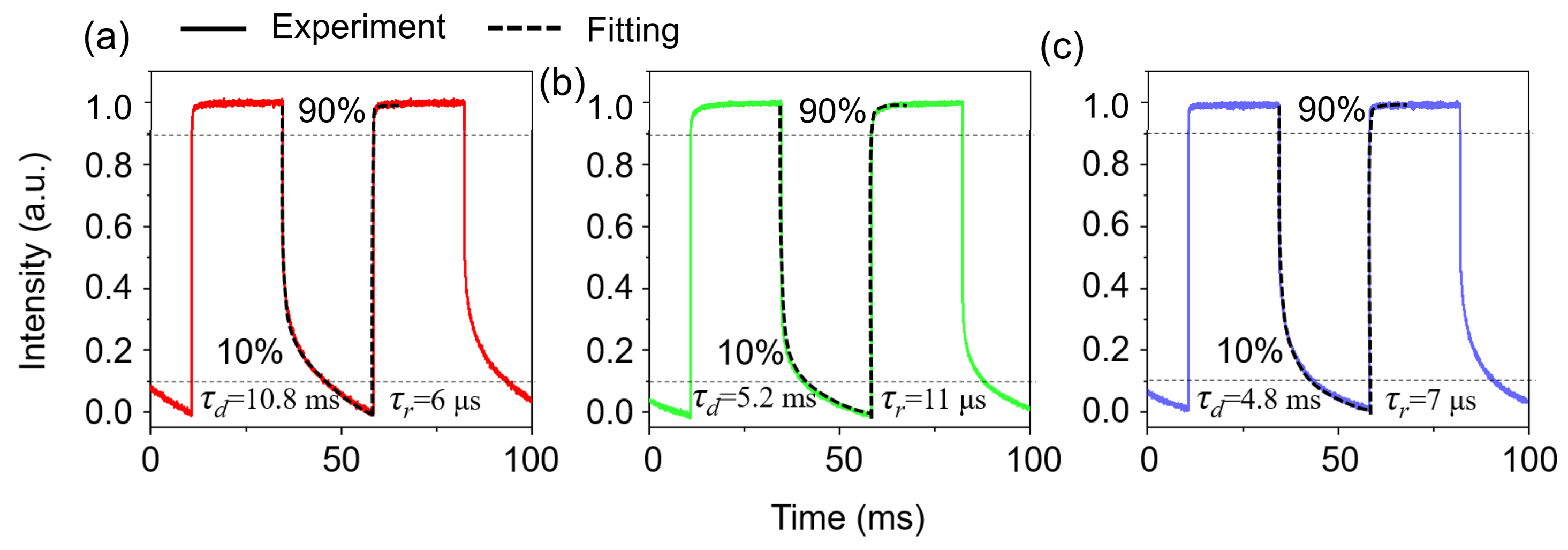


**Fig. S9.** Experimental and fitting data of transient response to calculate rise and decay time of the perovskite PDs (a is red PD, b is green PD, c is blue PD). The fitting equation is represented as: *I=I_0_+Ae^-t/τ1^+Be^-t/τ2^*, where, *I_0_* is steady-state photo-current, *A* and *B* is maximum amplitude of exponential constants, *t* is the time and *τ_1_* and *τ_2_* are the time constants in association with the exponentials.

**
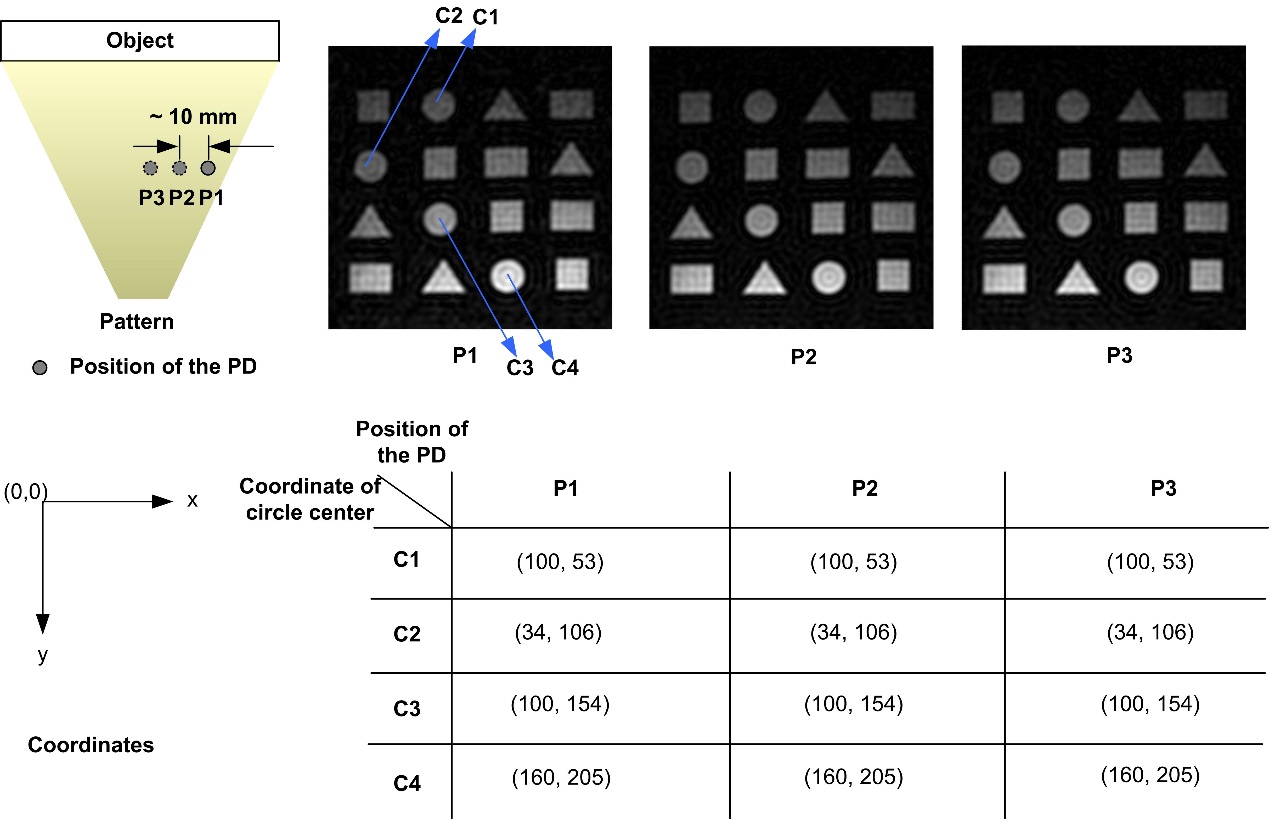
**

**Fig. S10.** The influence of PD position on imaging results.


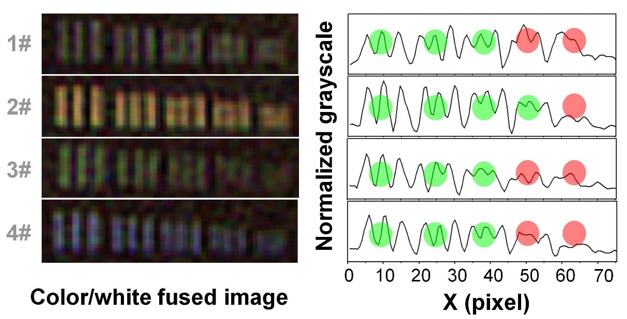


**Fig. S11.** The fusion color/white image and its normalized grayscale of 1-4#.

**Table S1.** Comparison of photodetection performance of perovskite-based photoreceptors.


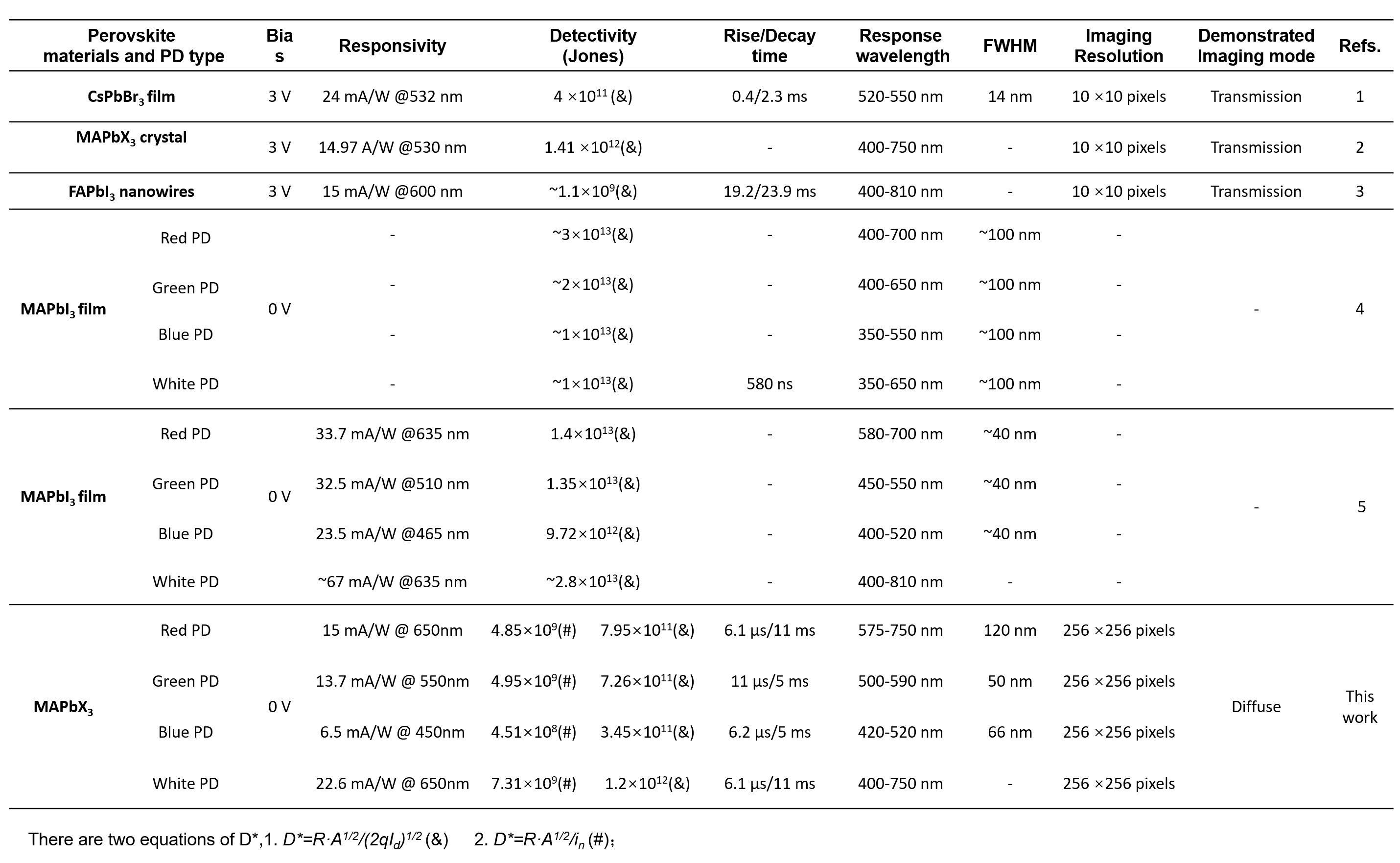


PD: photodetector, FWHM: full width at half maximum

**References:**

1. Noh, J. H. et al. Chemical management for colorful, efficient, and stable inorganic-organic hybrid nanostructured solar cells. Nano Lett. **13**, 1764-1769 (2013).
2. Wang, Q. et al. High-resolution, flexible, and full-color perovskite image photodetector via electrohydrodynamic printing of ionic-liquid-based ink. *Adv. Funct. Mater.* **31**,2100857 (2021).
3. Gu, L. et al. A biomimetic eye with a hemispherical perovskite nanowire array retina. Nature 581, 278-282 (2020).
4. Tsai, W.-L. et al. Band tunable microcavity perovskite artificial human photoreceptors. Adv. Mater. 31, 1900231 (2019).
5. Lu, J.-H. et al. Perovskite photosensors integrated with silver resonant-cavity color filters display color perception beyond that of the human eye. *Adv. Funct. Mater.* **30**, 2002503 (2020).
